# COVID-19 and municipal solid waste (MSW) management: a review

**DOI:** 10.1007/s11356-021-13914-6

**Published:** 2021-04-20

**Authors:** Atanu Kumar Das, Md. Nazrul Islam, Md. Morsaline Billah, Asim Sarker

**Affiliations:** 1 Department of Forest Biomaterials and Technology, Swedish University of Agricultural Sciences, SE-90183 Umeå, Sweden; 2 Forestry and Wood Technology Discipline, Khulna University, Khulna, 9208 Bangladesh; 3 Biotechnology and Genetic Engineering Discipline, Khulna University, Khulna, 9208 Bangladesh; 4 Umeå International School of Public Health, Umeå University, SE-90187 Umeå, Sweden

**Keywords:** Municipal solid waste (MSW), COVID-19 pandemic, MSW management strategy and challenges, Occupational health

## Abstract

Municipal solid waste (MSW) represents an inevitable by-product of human activity and a major crisis for communities across the globe. In recent times, the recycling of MSW has drawn attention as the process can add value through resources from the recovered waste materials and facilitates the process of circular economy. However, during the unprecedented coronavirus (COVID-19) outbreak, the risk of infection with the highly contagious virus has proven detrimental to the continuation of MSW as a valuable resource. The volume of waste, especially household waste, is higher; face masks, PPE (personal protective equipment), and hazardous materials such as batteries and empty chlorine bottles are examples of extra waste that have arisen during the pandemic. Various countries have set up initiatives for MSW management, including safety measurements for employees in the MSW management sector. The use of disinfectant prior to sorting waste, as well as storing waste for 9 days, may help to inactivate the COVID-19 virus, ensuring an appropriate safety level for MSW management. This work aimed at studying different MSW management strategies, specific challenges, and possible solutions for better understanding for those involved in waste management, in addition to providing a possible management strategy during and post-COVID-19 pandemic.

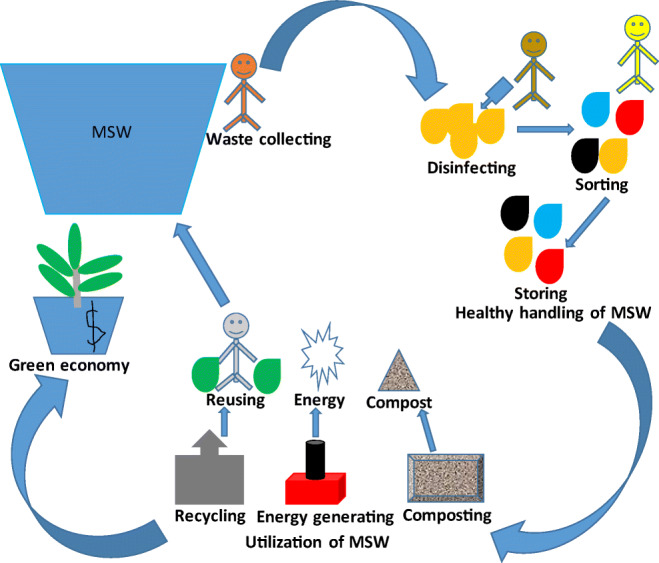

## Introduction

The constant ecological and environmental degradation has necessitated the transfer of global economic growth to green growth for sustainable development (Abu Hajar et al. 2020; Bina 2013; Luukkanen et al. 2019; Wanner 2015). However, municipal solid waste (MSW) management remains an environmental concern and one of the major constraints for green growth across the globe (Iqbal et al. 2020). MSW includes waste generated in households within a community under a municipality and waste generated from industrial or commercial sectors. The trend indicates that the amount of MSW is predicated to increase globally by 2.3 billion tonnes by 2025 (Hoornweg and Bhada-Tata 2012) and 3.4 billion tonnes by 2050 (Kaza et al. 2018). Estimates show that cooperative economic organizations and developed countries are responsible for the generation of half of the total MSW produced globally (Lu et al. 2020). The organic content of global MSW is 46% but rises to 64% in developing countries (Foundation 2017; Lu et al. 2020). Landfill remains the most economical method of disposal worldwide (Renou et al. 2008); 70% of MSW is disposed of as landfill and 19% is recovered for recycling and composting (Abylkhani et al. 2020). Recycling and other alternative uses, i.e., energy and compost, constitute appropriate practices for MSW management, with the potential to achieve a greener economy that encompasses sustainable development. A proper method for sorting and disposal of MSW may be the main goal of MSW waste management.

Nevertheless, MSW management is hazardous to health, especially for workers who are exposed to waste during handling (i.e., sorting, transportation, and recycling) in the event of an epidemic or pandemic. In recent years, the outbreak of severe acute respiratory syndrome coronavirus-2 (SARS-CoV-2) and the subsequent global COVID-19 pandemic have necessitated scrutiny of the MSW management practices and approaches in various countries. MSW management requires effective participation, coordination, and concerted efforts toward efficient delivery among various interconnected bodies, as it involves waste generation, waste composition, collection, recycling, pre-treatment and treatment, and finally disposal. These management aspects differ in terms of the legal, economic, governmental, political, administrative, and environmental players in different countries. Management structure and function can be country-specific, demand-driven, or necessity-oriented and rely on socioeconomic, behavioral, cultural, institutional, and political frameworks. Therefore, MSW management involves multi-professional drivers, and at times, the failure of one component can lead to the collapse of the whole management strategy (Pharino 2017).

COVID-19 has caused unprecedented havoc and distress in human life across the globe (Mol and Caldas 2020). The causative virus has been reported to persist on different surfaces for periods ranging from 3 hours to 9 days (Kampf et al. 2020; van Doremalen et al. 2020; Wang et al. 2005). The transmission of COVID-19 virus occurs through contact and the respiratory system, and the main sources of respiratory droplets are coughing and sneezing by an infected person (WHO 2019). The long persistence of this virus on inert surfaces and human-to-human transmission (Chan et al. 2020) has resulted in the rapid spread of COVID-19 virus. Thus, there is a possibility of workers in the MSW management sector being infected with COVID-19 virus and thus contributing to community transmission, since they are directly exposed to contaminated waste generated from infected people. This has necessitated the installation of appropriate protection measures for workers during the COVID-19 pandemic, as waste pickers, doctors, and nurses have all been key workers throughout this period (covid-19-Jordan 2020). Different countries have adopted different initiatives to address this issue through training, policy, and monitoring practices (covid-19-Jordan 2020; covid-19-Lebanon 2020), but the major risk factors have yet to be appropriately overcome at a global level. Effective management of MSW during the COVID-19 pandemic can contribute to circular economy, since it will allow for recycling and conversion to valuable purposes, i.e., energy, rather than landfill.

Properly treated MSW can be used for recycling and production of valuable products, since the waste is disinfected during proper treatment. Biodegradable waste can be used for production of products such as methanol, ethanol, butanol, etc., as well as for bioenergy, i.e., bioelectricity, biomethane, biohydrogen, etc. Meanwhile, nonbiodegradable waste can be used for pavement and construction processes (Malinauskaite et al. 2017). Therefore, when appropriate treatment methodologies are adopted, different types of waste can be utilized based on their origin and composition (Velvizhi et al. 2020); this strategy can offer opportunities for resource utilization, its subsequent optimization, and the sustainable economic development of a country (Falcone et al. 2020; Fan et al. 2020; Velvizhi et al. 2020).

However, there have been some studies on country-based MSW management during the still-ongoing COVID-19 pandemic (Torkashvand et al. 2021; Belhadi et al. 2020; Ikiz et al. 2021; Ismail et al. 2020; Kulkarni and Anantharama 2020; Lima et al. 2020; Mihai 2020; Penteado and de Castro 2021; Ragazzi et al. 2020; Yang et al. 2021; Zand and Heir 2020). These studies show that different countries have adopted and adhered to different strategies based on their context; no comprehensive study of MSW management strategies adopted during the pandemic has yet been attempted. Therefore, in this review, we tried to explore the MSW management strategies of various countries and their challenges in the context of MSW management, introduce possible solutions, and incorporate the concept of circularity during management as circular economy has the potential to boost the entire economy by maximizing resource utility. In addition, we discussed the general and additional types of MSW generated during the pandemic.

## Composition of MSW and transmission possibility of COVID-19 virus through MSW

MSW comprises a pool of different types of solid waste generated by different household activities by people living in a community, either in an urban or rural setting, where there is likelihood of direct physical contact between people. Therefore, transmission of the COVID-19 virus can occur through generation of MSW and its subsequent management. A brief description of the general composition of MSW and the transmission mechanics of the COVID-19 virus through the surfaces of MSW are presented in this section.

### Amount and composition of MSW during COVID-19 pandemic

During this pandemic, it has been possible for the amount and type of waste generation to increase or decrease depending on the location (Naughton 2020). The reasons are twofold; on the one hand, major businesses and schools, which usually generate large volumes of waste, have remained closed for a certain period. On the other hand, for the businesses that remain open, household, medical, and agricultural waste generation has increased substantially. It has been observed that the amount of MSW has increased by 3.3% and organic waste by 13.3% within a year in New York City alone (Staub 2020). However, in Hubei Province, China, the amount of MSW in large- and medium-sized cities has reduced by 30%, but the generation of medical waste (infectious and non-infectious) has increased by more than 370% (Klemeš et al. 2020). More than 80% of total healthcare waste is non-infectious and needs to be collected and disposed as MSW (WHO 2020a, b, c, d). Thus, the composition of MSW is heterogeneous. The general overview of this MSW composition, including waste that has been newly added during the COVID-19 pandemic, is presented in Table 1. The composition of MSW generated from different sources has remained similar during the pandemic. However, the quantity and components of waste may vary. It has been observed that the composition of MSW is dynamic; within each community, it is influenced by several factors such as income level, lifestyle, season, household type, level of affluence, and location. In general, developing countries and rural areas generate more organic waste, i.e., food waste (Lu et al. 2020). In contrast, a larger amount of metals, plastics, and glass are generated from high-income households because of their consumption of processed foods and different commodities. Overall food waste during the COVID-19 pandemic has increased by 12% (Aldaco et al. 2020).

**Table 1:** Types and composition of MSW

MSW generated during normal situation		
Type of wastes	Composition of wastes	References
Organic	Kitchen waste, food waste, garden waste	Edjabou et al. (2018), Sahimaa et al. (2015), Zorpas et al. (2015)
Plastic	Pure plastic, plastic film, packaging, bottles	Edjabou et al. (2018), Lombardi et al. (2010), Sahimaa et al. (2015), Zorpas et al. (2015)
Glass	Packaging and non-packaging glass	Aja and Al-Kayiem (2014), Bernache-Perez et al. (2001), Lombardi et al. (2010), Miezah et al. (2015), Sahimaa et al. (2015), Zorpas et al. (2015)
Metal	Packaging	Lombardi et al. (2010)
Hazardous material	medicines, batteries, accumulators, and others	Lombardi et al. (2010)
Additional MSW generated during COVID-19 situation		
Plastic	Masks, hand gloves and empty bottles of disinfectants, test kits, boots, ear protectors	ISWA-Lebanon (2020), ISWA-Uganda (2020), Penteado and de Castro (2021)
Glass	Empty bottles of disinfectants, Glass ampoules and glass containers of vaccines	Kulkarni and Anantharama (2020)
Cloth	Masks, PPEs (Personal protective equipment)	Penteado and de Castro (2021)

Organic waste includes kitchen waste (Sahimaa et al. 2015), food waste (Edjabou et al. 2018; Zorpas et al. 2015), and garden waste (Edjabou et al. 2018; Sahimaa et al. 2015). Kitchen waste includes the skins of fruits and vegetables (Sahimaa et al. 2015). Rotten vegetables are also considered as kitchen waste. Food waste includes fish, meat, cooked food, bakery and dairy products, fruits, and coffee grounds (Edjabou et al. 2018; Zorpas et al. 2015). Used tea bags and tea grounds are categorized under food waste. Gardening waste includes flowers (Edjabou et al. 2018), sticks, and branches (Sahimaa et al. 2015).

The main components of MSW paper are cardboard, card, board, regular paper, and other paper. Cardboard is categorized into packaging, non-packaging, laminated with aluminum, laminated without aluminum, and stiff cardboard materials (Bernache-Perez et al. 2001; Lombardi et al. 2010). Card is subdivided into liquid cartons and card (i.e., greetings cards, invitation cards, and card such as clothing and luggage tags) (Pan and Voulvoulis 2007). Beverage cartons, corrugated boxes, and folding boxes fall under the category of board. Regular paper includes books, booklets, journals, phonebooks, office paper (Edjabou et al. 2018; Miezah et al. 2015), wrapping paper, photographic paper (Lombardi et al. 2010), newsprint, high-grade paper, corrugated paper (Aja and Al-Kayiem 2014; Lombardi et al. 2010), newspaper (Edjabou et al. 2018; Lombardi et al. 2010; Miezah et al. 2015; Pan and Voulvoulis 2007; Zorpas et al. 2015), magazines (Edjabou et al. 2018; Pan and Voulvoulis 2007; Zorpas et al. 2015), and advertisement leaflets and posters (Edjabou et al. 2018, Zorpas et al. 2015). Other paper includes toilet paper (Zorpas et al. 2015), feminine hygiene pads (Bernache-Perez et al. 2001), tissue paper (Miezah et al. 2015), kitchen tissues/towels (Zorpas et al. 2015), and miscellaneous papers (Aja and Al-Kayiem 2014; Edjabou et al. 2018; Pan and Voulvoulis 2007). Packaging waste, especially paper-based material, has increased substantially due to spikes in online shopping, as many countries have adopted lockdown measures that have shut down over-the-counter and physical shopping during the COVID-19 pandemic (covid-19-Austria 2020).

Plastic waste comes in different forms, i.e., packaging and film (Aja and Al-Kayiem 2014; Sahimaa et al. 2015; Zorpas et al. 2015). Packaging plastics include garbage bags, packaging materials, high-density polyethylene (HDPE), low-density polyethylene (LDPE), polyethylene terephthalate (PET), and other plastics (Aja and Al-Kayiem 2014; Edjabou et al. 2018; Lombardi et al. 2010; Sahimaa et al. 2015; Zorpas et al. 2015). Plastic film is categorized into the forms of pure and composite plastics used for wrapping (Edjabou et al. 2018). Plastic bottles are also considered plastic waste (Zorpas et al. 2015). These include empty containers for cooking oil, soft drinks, honey, and water. The aforementioned sharp increase in online shopping due to COVID-19 has also significantly contributed to the generation of a large volume of waste from plastic packaging material (covid-19-Austria 2020).

Glass and different types of material prepared from glass are an indispensable part of human life and are widely used. Glass is classified as either clear or stained glass (Aja and Al-Kayiem 2014; Bernache-Perez et al. 2001; Lombardi et al. 2010; Miezah et al. 2015). Both types of glass are divided into packaging and non-packaging glass (Bernache-Perez et al. 2001; Sahimaa et al. 2015).

Metal containers are used for preserved green vegetables, soft drinks, milk, cookies, etc. in order to increase shelf life for perishable goods and items. These containers are used in considerable quantities every day. However, some of them are designated as hazardous MSW, which need special considerations for disposal and reuse. For example, medicines, batteries, accumulators, and other related hazardous materials fall into this class (Lombardi et al. 2010). Used containers for liquid chlorine and sanitizer constitute newly added MSW from the COVID-19 pandemic.

MSW may contain different pathogens, arising from items such as disposable diapers—it has been found that more than 10% of fecal soiled disposable diapers in landfills contain enteroviruses. Another primary source of pathogens is sewage biosolids, where co-disposal is practiced. Both food and domestic pet waste contain varying levels of pathogens. Biosolids are a by-product of physical (primary treatment), biological (activated sludge), and physicochemical precipitation of suspended solids (by chemicals) in the treatment processes adopted for waste management.

The outbreak of COVID-19 has resulted in the use of personal protection equipment (PPE), i.e., masks and rubber gloves used by the general population (covid-19-Belgium 2020); these new types of waste have added to the quantity of MSW. Though the face masks, tissue papers, and wipes generated by confirmed or suspected coronavirus-infected individuals are MSW in nature, they are now considered as medical waste (ADB 2020). For a better understanding of the MSW generated during the COVID-19 pandemic, we have included these waste types as additional MSW generated during the pandemic (Table 1).

### Survival rate of COVID-19 virus on substrate and its transmission

**Fig. 1:**
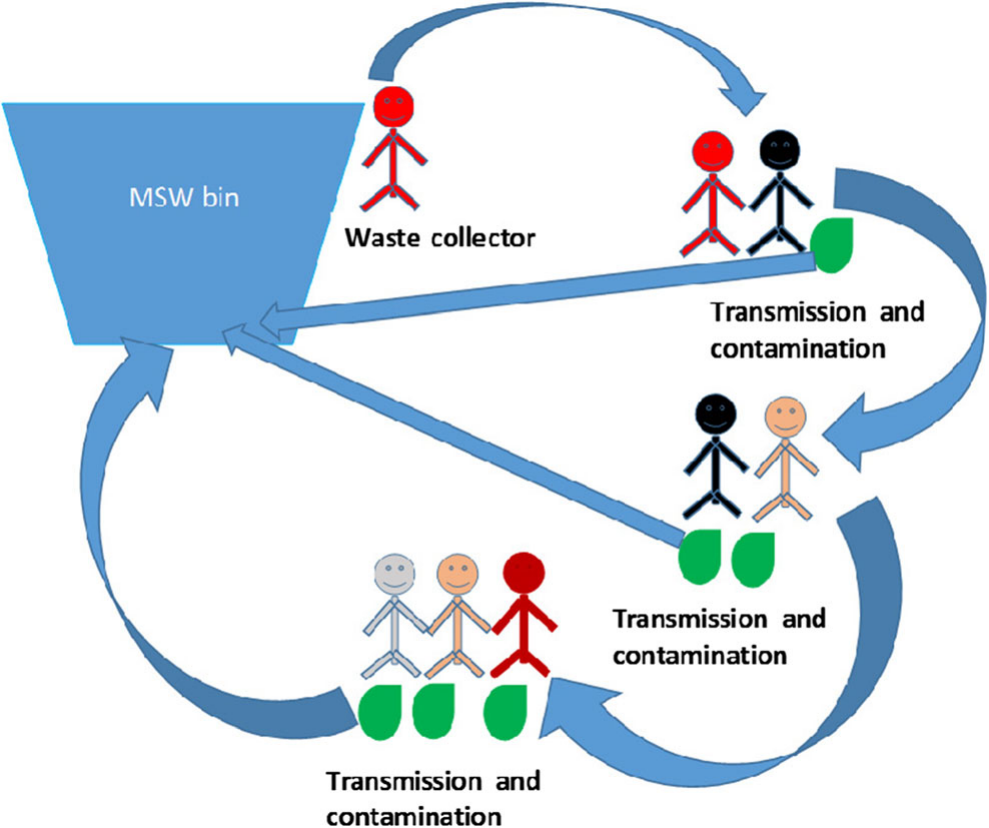
Transmission of COVID-19 virus through MSW (adopted from Das et al. 2021)

The transmission of COVID-19 virus occurs through sneezing, coughing, contact with COVID-19-contaminated surfaces, and physical contact with COVID-19-infected individuals (Burke et al. 2020; Chan et al. 2020; Ghinai et al. 2020; Hamner et al. 2020; Huang et al. 2020a; Huang et al. 2020b; covid-19-Netherlands 2020; Liu et al. 2020; Pung et al. 2020; WHO 2020b). The information regarding the survival rates of the COVID-19 virus on different substrates is crucial for devising an effective management approach for MSW. Researchers have determined that the survival rate ranges from a few hours to a few days based on the type of substrate (Kampf et al. 2020; van Doremalen et al. 2020; Wang et al. 2005). The survival rate of the virus post-aerosolization on copper material, cardboard, plastic, and stainless steel are 3 h, 4 h, 24 h, and 2–3 days, respectively (van Doremalen et al. 2020). Other researchers have reported that it can remain on inanimate surfaces such as metal, glass, and plastic for up to 9 days (Kampf et al. 2020). Other types of coronaviruses are reported to persist in dechlorinated tap water and hospital wastewater at 20°C for 2 days (Wang et al. 2005). The long survival rate of COVID-19 virus on substrate may cause a higher spread of the virus due to the frequent use of these materials and generation of waste in daily life. The possible transmission of the COVID-19 virus through waste is presented in Fig. 1. However, the persistence of the COVID-19 virus and its survival rate on other surfaces such as wood, cloth, etc. are yet to be determined; this information would help to devise an effective management approach for MSW.

## Treatment and disposal of MSW during the COVID-19 pandemic

**Fig. 2:**
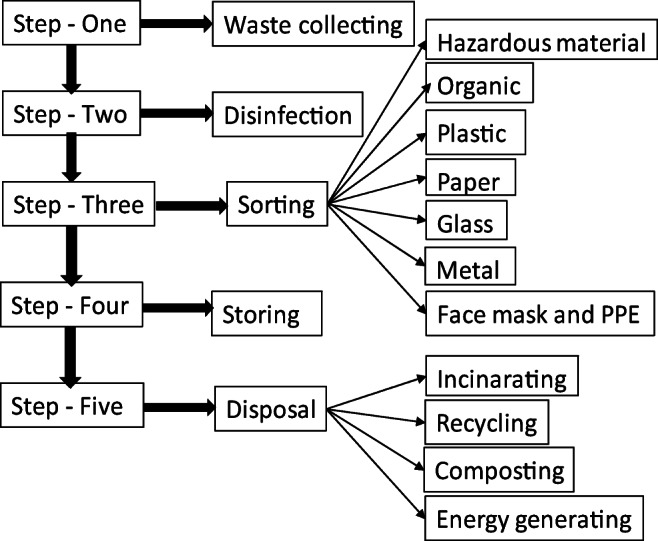
Pathway of MSW handling during COVID-19 pandemic

MSW collectors and employees involved in sorting, treating, transporting, and final disposal are directly exposed to coronavirus-contaminated waste. Improper treatment of MSW and lack of appropriate PPE may lead to COVID-19 virus infection among employees dealing with MSW and consequently a rapid spread of the virus. Disinfecting, sorting, and storing waste for 9 days with proper safety measurements may reduce or stop the spread of the virus. Thus, this approach can facilitate safe MSW management and proper uses for the waste (covid-19-Lebanon 2020). The steps for handling MSW during the COVID-19 pandemic are explained in Fig. 2.

As per WHO guidelines for the COVID-19 pandemic, waste generated during home quarantine should be kept in black bags and closed prior to disposal and collection by the MSW management sector. It is recommended that used tissue papers and other materials for sneezing or coughing are disposed of in waste bins and that the hands are then sanitized with disinfectant (WHO 2020d). Face masks, tissue paper, and wipes generated by people quarantining at home during the pandemic are advised to be kept in yellow medical bags, which are considered as medical waste. The bags should be tied with the swan neck method and disinfected by spraying 1% bleach or 0.5% chlorine solution on the surface of the sealed bags. If there is no medical waste collection system, it should be stored for 72 h and disposed of by incineration or landfill as general MSW (ADB 2020).

The main practice for MSW disposal is landfill, due to its cost-effectiveness (Renou et al. 2008). Recycling and composting of MSW are also done, though in lesser percentages than landfill (Abylkhani et al. 2020). Among MSW, metal, paper, and plastic are recycled and incinerated, while glass can be fully recycled (Abu Hajar et al. 2020). Meanwhile, organic waste is used for compost and incineration (Abu Hajar et al. 2020; Lu et al. 2020). In India, around 60% of plastic is recycled (Alpizar et al. 2020); however, the pandemic has decreased the recycling of plastic in countries with smaller economies, and increased local burning strategies as an attempt to avoid virus contagion (Corburn et al. 2020). Though deploying mobile incineration facilities, countries with larger economies can overcome the negative effects of COVID-19 on plastic waste management. Compost and incineration as an MSW management strategy can reduce the burden of landfill (Abu Hajar et al. 2020). Incineration is the main system for MSW treatment in China (Chen et al. 2010; Lin et al. 2020). Though the COVID-19 pandemic has sometimes rendered MSW management difficulty and troublesome due to the increased risk of community transmission, an integrated MSW management strategy with appropriate treatment technology and containment could provide a sustainable and environmentally friendly option (Staub 2020).

## MSW management strategy taken by different nations

**Fig. 3:**
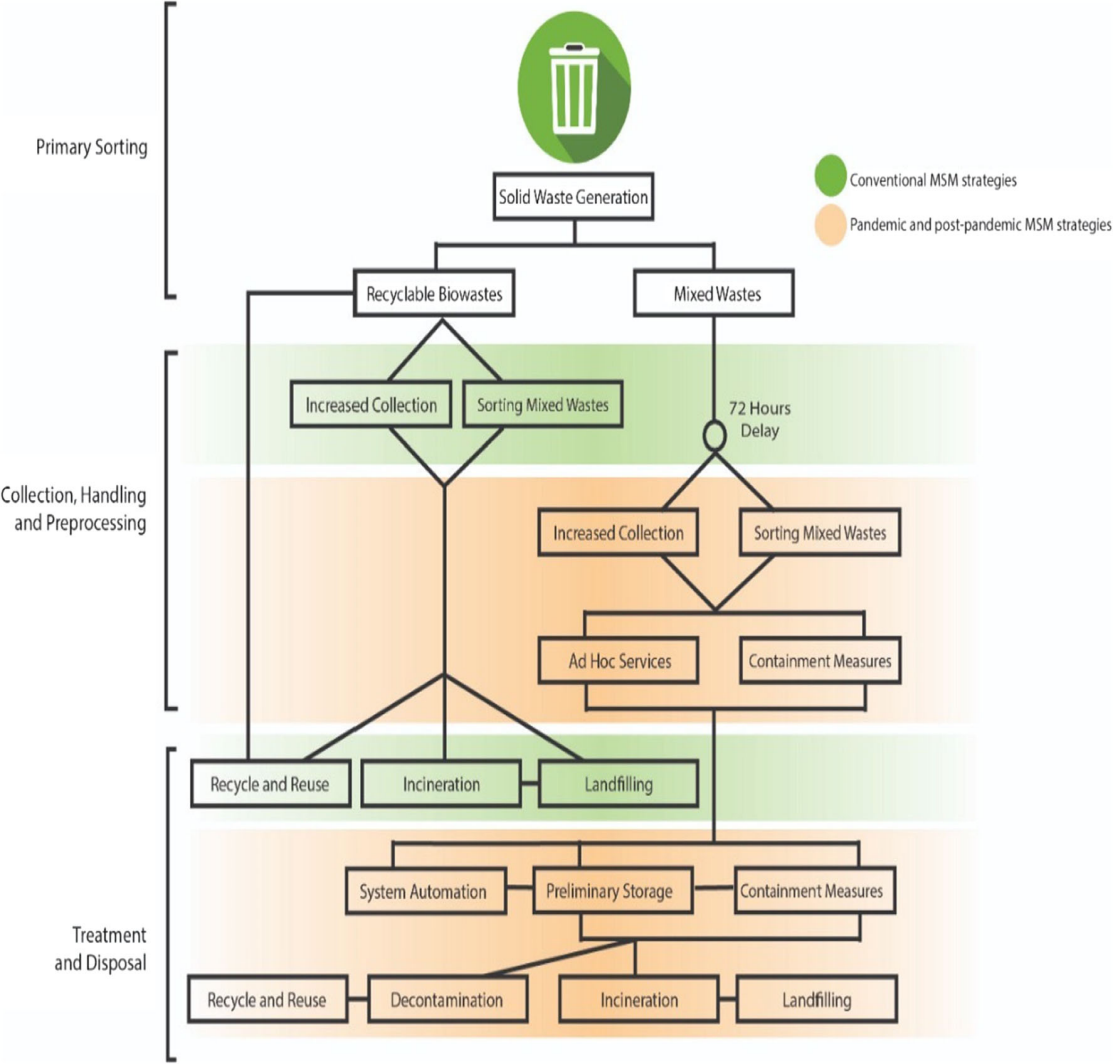
General strategy of MSW management during COVID-19 pandemic (adopted from ACR^+^ 2020)

While the COVID-19 pandemic is still ongoing, an improper MSW strategy may cause health risks for employees, especially waste collectors in the waste management sector (Cruvinel et al. 2019). This represents a large group of people involved in the collection, sorting, and sale of waste generated by a community. In addition, COVID-19 patients can generate a huge amount of virus-contaminated waste. An inefficient strategy can lead to infection among both MSW workers and the general population, leading to a rapid outbreak (Mol and Caldas 2020). Therefore, the general procedures of MSW management (Fig. 3), special waste treatment, and safety and health measures for employees in the waste management sector have become crucial for proper containment and implementing control measures to prevent further spread of the virus (ACR^+^ 2020).

However, different countries and continents across the globe have adopted varying strategies for MSW management during the COVID-19 pandemic in order to control the rapid spread of the disease. The important strategies adopted in different continents are presented in Table 2. We have discussed country-specific strategies within this section.

**Table 2:** Important strategies taken by different parts of world for managing MSW during COVID-19 pandemic (ISWA-COVID-19 2020)

Part of the world	Safety for employee	Waste collection	Special waste treatment	Face mask and PPE	Waste trade	Recycling	Public awareness
Europe	Suggested for using appropriate PPE	Restricted Number of employees; Maintaining social distance by employee	Thermal treatment	-	Among EU member countries	General public are not allowed	-
Australia	Suggested for using appropriate PPE	Normal	-	-	-	-	-
USA	Suggested for using appropriate PPE	Normal	-	-	-	-	-
Latin America	Suggested for using appropriate PPE; Laying off aged workers	Possible contaminated waste should put in double bag; Maintaining social distance by employee	-	Should put in separate bag for considering as medical waste	Importing is stopped	-	-
Middle East	Suggested for using appropriate PPE	Wastes generated by isolated people should seal in bag and treat as healthcare waste, Maintaining social distance by employee	Wastes are being disinfected before sorting; Sorted wastes are stored for 9 days before handing over	Waste PPE should be placed in the special and considered as hazardous waste	Wastes carried by ship are allowed to send outside for recycling	-	Facebook is using for public awareness to handle waste
Asia	Suggested for using appropriate PPE	Restricted Number of employees; Maintaining social distance by employee; Segregation should be done at source	-	Special bins are used for collecting	Import and export are banned	-	Social media and posters are using for public awareness to handle waste
Africa	Suggested for using appropriate PPE	Normal	-	No special care	-	-	Poster is using for public awareness to handle waste

### Europe

The quantity of household waste generation and incineration is imbalanced in Belgium (covid-19-Belgium 2020). People are asked to dispose of their waste daily to avoid bulk deposition of waste (covid-19-Greece 2020) and to close the containers or bags after disposal of the waste (covid-19-Belgium 2020). In the Netherlands, dry waste such as wastepaper and textiles is stored at home for as long as possible, and then is collected at a recycling location (covid-19-Netherlands 2020). Waste collectors are advised to follow certain precautionary measures, i.e., maintaining social distance, using safety gloves, sanitizing hands, and cleaning trucks regularly (covid-19-Belgium 2020; covid-19-Greece 2020). Textile waste is not collected as the business-related textile market has been halted due to the COVID-19 outbreak. Used face masks and PPE from households are considered as residual waste rather than biowaste (covid-19-Belgium 2020; covid-19-Italy 2020; Klemeš et al. 2020); people have been instructed to keep this waste in orange bags in England (covid-19-UK 2020). However, there are no special measurements in place for treating face masks in Sweden (covid-19-Sweden 2020). The authority of the MSW management sector in Belgium has advised scientists to investigate the possible risks of contamination from handling this waste (covid-19-Belgium 2020). Treatment options for MSW have included thermal treatment prior to sorting of waste to prevent further COVID-19 virus infection among waste pickers and the general population in Austria (covid-19-Austria 2020), while the incineration temperature is strictly maintained at 1000°C to ensure safe destruction of infectious waste materials in Germany (Klemeš et al. 2020). Recycling units have been kept open to maintain smooth MSW management in Belgium (covid-19-Belgium 2020), but are closed to the public in Greece and Germany due to COVID-19 restrictions (covid-19-Greece 2020; Kumar and Rasquin 2020).

The Swedish and French MSW management sectors are still adhering to the regular strategies practiced in normal MSW management (covid-19-France 2020; covid-19-Sweden 2020), but Sweden’s main strategy is to limit visits for handling bulky waste (covid-19-Sweden 2020), while France is aiming to sort waste properly (covid-19-France 2020). The amount of imported waste has decreased during the pandemic, and the extra domestic waste is being handled without any trouble in Sweden (covid-19-Sweden 2020). Shipment actors for waste have been advised to follow green guidelines by the European Union (EU) (Kumar and Rasquin 2020). In England, the frequency of waste collection is based on type of waste (covid-19-UK 2020), while in Italy, waste management follows WHO guidelines (covid-19-Italy 2020).

Most countries in the EU have instituted safety measurements for employees directly involved in waste management sectors. Thermal treatment of waste has been conducted in order to avoid the spread of COVID-19 virus. In addition, the EU has specified a path for transporting waste in order to manage it without any disturbance. Nevertheless, instructions for preventing re-infection and returning to the workplace for recovered employees are still not available.

### Oceania

In New Zealand, PPE has been provided for workers involved with MSW management so that they can maintain personal safety. The strategy for waste collection and disposal has been maintained as normal during the pandemic. However, landfills and waste transfer stations are only open for essential services. Public access to waste transfer stations is limited. Contaminated waste from households is considered normal waste for recycling (covid-19-New Zealand 2020). However, there is no available information about disinfecting MSW in New Zealand. The guidelines for preventing COVID-19 virus re-infection and returning to the workplace for infected employees have not yet been reported.

### North America

There is a clear COVID-19 message for workers involved in waste collection and recycling in the USA. Information on ways to prevent infection and reduce the spread of the virus has been provided to workers. Guidelines for returning to the workplace have been given to infected employees (CDC 2020). Waste collectors and recycling personnel are asked to use PPE, i.e., eye and face protection, and puncture-resistant gloves for avoiding exposure to contaminants. There are clear instructions for sanitizing the workplace and equipment. Treatment of MSW that either is contaminated or has the potential to be contaminated by COVID-19 virus has been made imperative (NWRA 2020). Safety measurements for employees handling MSW are taken into consideration, but the strategy for disinfecting MSW has not yet been explained.

### Latin America

In Latin America, food waste and packaging materials have increased due to more frequent food deliveries at home. Recyclable waste is being collected along with residual waste, following regular rules and regulations (covid-19-Latin-America 2020). In Brazil, the MSW management sector has taken different preventive measures by identifying at-risk workers, i.e., aged and sick individuals, and has subsequently laid off this at-risk group. Hand sanitizer has been provided to all waste operation units and vehicles, and the cabins of vehicles are cleaned before and after each work cycle. Possible contaminated waste should be packed using double bags; the waste bag should be put inside another bag for greater protection. The transport of MSW to other countries has been restricted due to the pandemic. Recycling centers are closed to the public in order to maintain social distance. It is suggested that used masks and PPE from households be put in separate containers rather than in waste bins (covid-19-Brazil 2020). Instructions for use of PPE by employees in the MSW management sector and procedures for disinfecting MSW are not clear based on the available information. In addition, Latin American countries have failed to devise any guidelines similar to those of the European Union (EU) for the export of MSW.

### Middle East

In Israel, the quantity of packaging waste has increased by 20% compared to pre-pandemic levels. Waste generated by households has increased substantially, as people have been staying at home due to lockdown measures and have consumed more during this period (covid-19-Israel 2020). The MSW management sectors of Israel and Jordan have provided guidelines to collect and transport waste using proper safety measures (covid-19-Israel 2020; covid-19-Jordan 2020); meanwhile, in Lebanon, the MSW management strategy for collecting, sorting, treatment, and disposal of waste is following Lebanese legislation and predetermined procedures. Residents are asked to put waste bags outside the door at a fixed time (covid-19-Lebanon 2020). Workers have been instructed and trained to use PPE, goggles or full-face masks, ultra-filtering masks, elastic leather gloves, shoes, and long-sleeved clothes with head coverings and filters in order to avoid infection with the virus. They have also been instructed to use sanitizer to clean their hands frequently and to sterilize safety equipment and vehicles in order to prevent COVID-19 transmission. MSW collectors are required to maintain an appropriate distance from each other and from residents during collection of waste (covid-19-Jordan 2020; covid-19-Lebanon 2020). Recyclable waste carried by ships is allowed to be exported from Israel for recycling, but not that carried by airplanes. The Israeli Ministry of Health has instructed recycling industries not to recycle beverage packages or bottles. Meanwhile, waste management sectors have used Facebook campaigns to promote using single products, separating products, using soap instead of wipes, and making efforts to reduce food waste through properly devised plans. Landfill has increased slightly in Israel (covid-19-Israel 2020). Waste generated from infected individuals is considered medical waste or sent to sanitary landfills (covid-19-Israel 2020; covid-19-Lebanon 2020). Used PPE is considered as hazardous waste, and the instruction is to keep it in special bags (covid-19-Jordan 2020, covid-19-Lebanon 2020). However, there are no special measures in place for collecting face masks and PPE in Israel (covid-19-Israel 2020).

In addition, some exceptional approaches have been taken by the Lebanese MSW management sector to avoid physical contact. They have adopted an online system for accessing personal information in order to carry out registration of waste collection schedules and MSW management premises. Sanitizer has been placed at the entrances of offices, and liquid soap and paper towels supplied and fitted in washrooms. Collection bins must be disinfected after emptying; waste is required to be disinfected prior to manual sorting. The sorted waste is categorized based on the type and date of sorting. It is advised that this sorted waste be retained in its facility for 9 days prior to being handed over for further processing (covid-19-Lebanon 2020).

### Asia

In Singapore, Malaysia, Japan, and India, MSW management workers are trained to use PPE, identify the symptoms of COVID-19, and maintain social distance (Fujii 2020; covid-19-India 2020; covid-19-Malaysia 2020; covid-19-Singapore 2020). Disinfectant is provided for use in vehicles and on equipment in Malaysia (covid-19-Malaysia 2020). In Singapore, waste collectors are supervised during collection of contaminated waste. Residents are advised to use chute system bins or bins on doorsteps to dispose of waste (covid-19-Malaysia 2020, covid-19-Singapore 2020). Meanwhile, doorstep collection has been halted in India (covid-19-India 2020). In Japan, the public has been advised to sort waste themselves in order to reduce the pressure on waste collectors (Fujii 2020). The import and export of waste have been banned outright in Singapore and Malaysia. However, it is possible to recycle the existing quantity of waste without any landfill or incineration (covid-19-Singapore 2020; covid-19-Malaysia 2020). There are no special processes for managing masks and tissue paper, but people in Singapore and Malaysia have been asked to dispose of these items properly (covid-19-Malaysia 2020, covid-19-Singapore 2020). In contrast, in India, face mask waste has been categorized as domestic hazardous waste while the pandemic is still ongoing (covid-19-India 2020). Techniques for safe removal and disposal of face masks and gloves in order to avoid contamination have been promoted through social media and posters in offices in Malaysia (covid-19-Malaysia 2020).

China has taken nationwide systematic action to manage MSW during the COVID-19 pandemic. Local governments have been asked to take the necessary steps and follow the guidelines for MSW management. In Suzhou and Bengbu, special waste bins have been installed to collect face masks. The used masks are collected and directly transported to waste treatment plants by designated personnel using vehicles, in order to avoid the spread of the virus. MSW management tools, facilities, and public areas are disinfected and sterilized four times a day. The MSW management sector has been advised to collect waste from the bins in a timely manner. Campaigns are being planned to increase awareness of managing MSW. In Shanghai, residents are requested to segregate household waste as wet and dry waste rather than as kitchen, recyclable, hazardous, and other wastes at origin. Overall, local authorities are urged to follow MSW management strictly (Mingyu 2020).

### Africa

In South Africa and Uganda, workers in the MSW management sector have been given hand sanitizer, gloves, and special masks (covid-19-South Africa 2020; covid-19-Uganda 2020). Brochures detailing the safety rules and hygiene procedures for COVID-19 have been placed on walls at workplaces in South Africa (covid-19-South Africa 2020). The public is asked to dispose the used face masks following the regular waste management strategy (covid-19-South Africa 2020, covid-19-Uganda 2020). For MSW waste that is not incinerated, the Howden System Incinerator and Converter Non-Burn Technology are used for management (covid-19-South Africa 2020). However, the report lacks information on the disinfection procedures and disposal approaches for MSW.

## MSW management approaches

Possible management approaches based on different literatures (Bank 1999; Phonphoton and Pharino 2019; Schübeler 1996; Singh et al. 2020a, 2020b; Zhu et al. 2008) have been summarized in this section; these approaches could be adopted during the COVID-19 pandemic.

### Governance

Waste management is a multi-faceted complex governance issue. The dimension of waste management often entails different forms of governance, from private individuals to multijurisdictional approaches. Self-governance is necessary when an individual is responsible for waste generated by their own actions or settings, while multijurisdictional approaches are necessary when movement of waste takes place within different geographic locations. However, the majority of waste management governance is somewhere in between these two approaches (Vallero 2019). The World Bank considers MSW management to be one of the key roles of local government within a country (Hoornweg and Bhada-Tata 2012).

MSW management has been considered the most important service of local government; it is provided on a city-wide basis in both low-income and middle-income countries. Therefore, within a city’s administrative structure, management of MSW draws the largest single budget from government and constitutes one of the largest employers within the city. In addition, collection of solid waste inevitably falls within the realm of local government. A city without effective waste management is rarely considered to be capable of managing more complex public services such as health, education, and transportation. Poor management of waste puts a huge strain on health, local and global environment, and the economy, because management without appropriate measures brings about additional expenses, increases suffering within a neighborhood, and deteriorates quality of life. In addition, as MSW is generated worldwide, improper management significantly contributes to global environmental problems through generation of greenhouse gases, e.g., methane, and public health issues. Therefore, due to the global nature of waste generation, governance should essentially include the aspects relating to an increased global product supply chain, urbanization practices, and opportunities for the recycling industry (Vallero 2019).

It can be seen that there is no universal approach that can accommodate every aspect of MSW management in order to provide the most efficient and effective solution for governments. Therefore, reliable services should ensure an inclusive waste management system for all stakeholders. On a small scale, local authorities are usually accountable for operations related to collection, recycling, composting, and general waste handling (Abdel-Shafy and Mansour 2018). Larger-scale settings, such as metropolitan waste management authorities, usually accept logistical support and resources in order to work in collaboration with local authorities. The highest scale jurisdictions usually determine standards and devise policies for effective MSW management, for example, imposing restrictions on disposal of hazardous waste, the amounts that can be released into the environment, definitions and classifications of hazardous and non-hazardous substances, and laws relating to incineration and other less centralized waste disposal approaches (Vallero 2019).

Though it can be seen that most local MSW management decisions have a low chance of bringing about large-scale devastating outcomes if made improperly, for example, choice of trucks, daily operations, or landfill protocols, they might create an overall lack of governance support systems in the long run (Bahauddin and Uddin 2012; Vallero 2019). In addition, there are some low-probability, high consequence events that might pose special challenges to local risk managers, such as environmental engineering decisions. Therefore, every problem requires clear and factual communications within the support structure (Vallero 2019). The success of MSW management depends on proper synchronicity among all stakeholders and working harmony at every level of governance; this dictates its overall reliability.

### Communication

Communication is one of the most important approaches for MSW management (Falcone et al. 2020; Paul and Bussemaker 2020; Waste-Management 2020). A communication gap may break down the chain of custody for MSW management strategies, whether during the COVID-19 pandemic or at other times. Smooth MSW management is made possible through proper communication among the waste generator, collector, treater, disposer, and recycler. However, developing countries often suffer from poor communication among these actors; the pandemic has interrupted this communication even further (Sinha et al. 2020). It has become imperative that documentation be faster during this crisis, which has entailed the necessity of online communication, meaning the use of soft copies (through email and other interactive platforms) rather than hard copies. Clear instruction about collection, disinfection, and disposal places, i.e., recycling, combustion, and landfill, should be available on web-based platforms. This can help to manage waste without any unnecessary prohibition during the pandemic. In addition, engaging with the community can play a pivotal role in effective and safe MSW management by reducing waste generation and allowing safe disposal of waste (Falcone et al. 2020).

### Accountability: risk, reliability, and resilience

Accountability is crucial for successful and reliable MSW management during this COVID-19 pandemic. For this reason, proper documentation, i.e., type of waste, collector, transporter, and disposal location for the MSW, is of the utmost importance (Kulkarni and Anantharama 2020). Agreement between the waste management sector and the end user, i.e., the recycling or landfill actor, can allow reliable and smooth waste management. Waste vehicles should be marked based on waste type for clear understanding, and segregation should be performed properly to avoid co-mingling waste. There should be a proper system for reporting to the respective department after disposing of the waste. Mixing up waste and wrong documentation can lead to disposal in the wrong places, which may increase the risk of spreading the COVID-19 virus (Di Maria et al. 2020). Supervisors in the waste management sector should work together in order to ensure effectiveness and functionality of management techniques; through their respective authority, they can control MSW management properly during the pandemic.

## Challenges in MSW management

Due to the ongoing pandemic, MSW management poses great challenges in terms of waste treatment and safety requirements (ACR^+^ 2020). The quantity of MSW has increased substantially for public service providers due to lockdown and other measures for preventing community transmission (covid-19-Austria 2020); however, the recycling of waste has reduced during this pandemic (Zambrano-Monserrate et al. 2020). On the other hand, the use of virgin packaging material has increased because the recycling of packaging materials has been hampered during the pandemic (Kumar and Rasquin 2020). Household waste has increased by 20–30% due to food consumption at home and home shopping during lockdown. Gardening and bulky waste has also increased due to spring cleaning and gardening by people staying at home (covid-19-Malaysia 2020).

Infected people at home generate contaminated waste, which in turn is responsible for further infection and spread of the virus (Mol and Caldas 2020). As a result, workers in waste sectors have become more vulnerable and constitute a high-risk group for further infection (covid-19-Belgium 2020). Inappropriate management techniques and lack of protective equipment have further deteriorated MSW management in developing countries (Mol and Caldas 2020). At the same time, waste from plastic packaging and textiles has been rendered difficult to process, as the demand for recycled products has declined in some countries (covid-19-Netherlands 2020).

The management of used face masks and PPE by the general public has constituted a new challenge due to their recent inclusion, in substantial amounts, in waste. This has revamped the need for research in the MSW sector to formulate clear guidelines for management of this type of waste (covid-19-Belgium 2020). Most PPE is plastic-based material; it has been an enormous challenge to handle this unprecedented waste, which has been generated by the public through irrational and unregulated use rather than by healthcare workers (covid-19-Asia 2020).

During the COVID-19 pandemic, people have adopted various measures such as liquid chlorine, alcohol, and sanitizer to prevent the spread of infection at home. Unregulated use of corrosive chemicals has constituted a public health concern and, in some cases, has the potential to integrate into aquatic ecosystems and disrupt food chains.

After treatment, significant quantities of the pathogens present in raw sewage often remain in biosolids. On a volume basis, the concentration of pathogens in biosolids can be high because of adsorption of infectious agents, especially viruses. In addition, most microbial species present in raw sewage become concentrated in sludge during primary sedimentation. Although enteric viruses are too low in mass to settle by themselves, they also become concentrated in sludge because of their strong binding affinity to different particulate materials in sludge (Gerba and Pepper 2009). This may be the reason for the spread of the COVID-19 virus as well.

## Possible solutions

The overall composition of MSW dictates how the waste can be recycled. Organic waste can be utilized for production of compost as cheap fertilizer, while plastic and non-perishable waste can be recycled and reused (Ayilara et al. 2020; Sayara et al. 2020). Waste management becomes more efficient when it can cover the maximum area within a community. However, ensuring this maximal coverage requires a substantial level of resource allocation and policy integration (McAllister 2015; Abdel-Shafy and Mansour 2018). If waste can be subjected to circular economy, it can be transformed into valuable resources (Velenturf et al. 2019). Nevertheless, during the COVID-19 pandemic, the composition of MSW has varied considerably, which has required adoption of appropriate management strategies congruent with infectious material management (Ragazzi et al. 2020; Kulkarni and Anantharama 2020).

Waste should be treated in a sustainable manner without affecting human health or environment (Elleuch et al. 2018). The sorting of waste at home and disposal in appropriate bins can allow effective and safe handling of MSW (UNEP-IETC 2020). Waste collectors should first use PPE to prevent themselves from becoming infected. Waste should be disinfected prior to sorting, and then the sorted waste should be stored for 9 days before sending it for recycling or to be converted into energy (WtE). This strategy may help to stop further the spread of the COVID-19 virus (Sharma et al. 2020).

Incineration can destroy the virus and other pathogens due to the high temperature. Waste can be put in a bunker using an overhead crane, which can help to avoid human contact with the contaminated waste. It is then possible to feed the waste into the furnace using a feeding chute. Thus, the spread of the virus can be avoided. At present, while the COVID-19 pandemic is still ongoing, non-contaminated waste such as paper should be recycled whenever possible, after proper sorting. Non-recyclable and contaminated waste must be used for WtE (CEWEP 2020). Pyrolysis or WtE can offer another alternative way to manage MSW rather than sending it to landfill (Perrot and Subiantoro 2018).

The EU has set out an initiative to secure the continuity of waste shipments among its member states, following EU safety measurements, during the COVID-19 pandemic. The guidance is to avoid cross-border obstacles to movement of waste between EU countries (Waste-Shipment 2020).

Used batteries from different tools and equipment, used face masks, gloves, tissue paper, and other waste generated from infected persons should be separated as hazardous waste at source in order to reduce the risk for the MSW management sector (Lin et al. 2020). The extent to which the COVID-19 virus is present in wastewater is poorly understood, and its presence in an infectious form is yet to be determined (Kataki et al. 2021). Most previous reports on virus-related research in the biosolids field have focused on enteroviruses. Work by Gundy et al. (2009) showed that SARS-CoV-1, a relative of the COVID-19 virus, displays less environmental persistence in primary and secondary effluent compared to poliovirus. For sewage sludge, treatment by anaerobic or aerobic digestion and/or dewatering can reduce the numerical population of disease-causing agents, such as the COVID-19 virus, in biosolids. These treated biosolids can be used for the valuable purpose through hydrothermal liquefaction.

## Circularization of MSW management during the COVID-19 pandemic

Waste can be subjected to circular economic practices by transforming it into valuable resources through reusing, recycling, and repurposing (Parajuly et al. 2020). As we gradually attain a better understanding of the economic ramifications of the COVID-19 pandemic, the ways in which a circular model can contribute to the overall resource management system and recycling are slowly being unveiled (Ibn-Mohammed et al. 2021). The Investor Agenda group urges local governments to avoid the prioritization of risky, short-term emissions-intensive projects and instead focus on long-term low carbon alternatives in resource management and circularization of post-disaster recovery. The importance of these strategies has notably been highlighted in the USA, where several state treasurers have urged ventilator makers to make service manuals and repair-related resources available to help hospitals deal with the crisis. Similar integrated approaches could allow the use of COVID-19-associated overproduced and linear goods in a circular model incorporating public health, medical technology, engineering, and waste management (Blério 2020; Foundation 2017).

Another domain in which the circular economy appears particularly relevant is the highly sensitive area of food production and distribution. With traditional supply chain systems leaving open ends in terms of accumulating waste in both farms and households, closed circular food waste recycling can provide better alternatives (Giudice et al. 2020). Closed circular and regenerative practices can reduce the load of biodegradable and chemical wastes on management systems, meaning that more resources can be allocated to handling medical waste. As the Ellen MacArthur Foundation’s research has highlighted, a circular scenario could lead to a 50% reduction in pesticide and synthetic fertilizer use by 2030 in Europe compared to 2012 levels (Blério 2020). Finally, regenerative agriculture is also a powerful force in the climate crisis mitigation arsenal, as circular economy strategies could reduce emissions by 5.6 billion tonnes of CO_2_, corresponding to a 49% reduction in the projected total food system waste for 2050 (Foundation 2017).

In summary, the circularization of production and supply was gaining momentum even prior to COVID-19; in a complex and dynamic municipal setup, linear systems are little to nonfunctional and require closed circuits to mitigate pandemic and post-pandemic waste accumulation and demand for safe and efficacious management strategies.

## Conclusions

The highly contagious and persistent COVID-19 virus may infect MSW workers due to direct exposure to waste with poor safety measures. Extra waste volume due to medical waste generated from household during COVID-19 outbreak is creating troublesome for the waste management sector. The usage of appropriate PPE and safety measures for MSW workers is a top priority for every country. Proper treatment of MSW can result in providing a valuable source of energy and hence sustainable development. Each nation urges to follow the right procedures along with WHO guidelines to manage MSW properly with proper communication and accountability. Clear guidelines for handling household medical wastes in addition to health education about disinfection and management of MSW with scientific background are needed.
